# Multisensory environmental perception profiles and perceived restoration in an urban park during transitional seasonal conditions

**DOI:** 10.3389/fpubh.2026.1833501

**Published:** 2026-07-14

**Authors:** Bo Wang, Mengjia Chen, Lei Shi, Yang Cao, Yi Yang, Jia Yang, Shaobo Liu, You Peng

**Affiliations:** 1 School of Architecture and Art, Central South University, Changsha, China; 2 Sustainable Urban Development Lab, National University of Singapore (Suzhou) Research Institute, Suzhou, China; 3 Department of Architecture, College of Design and Engineering, National University of Singapore, 4 Architecture Drive, Singapore, Singapore

**Keywords:** latent profile analysis, multisensory environmental perception, perceived restoration, positive and negative affect, urban park

## Abstract

**Introduction:**

Urban environments expose individuals to complex multisensory stimuli that jointly influence psychological experience and wellbeing. Although previous studies have examined thermal, visual, and acoustic factors separately, less is known about how these sensory dimensions are jointly perceived and associated with perceived restoration. This study investigated multisensory environmental perception patterns and their association with perceived restoration in an urban park during the autumn-winter transitional season.

**Methods:**

A field survey involving 261 participants was conducted in a lakeside urban park in Changsha, China. Subjective evaluations of thermal, visual, and acoustic comfort, perceived restoration, and affective responses were collected simultaneously with in situ environmental measurements. Latent profile analysis (LPA) was applied to identify multisensory perception patterns, and multiple linear regression was performed to examine their associations with perceived restoration while controlling for affective, demographic, and environmental variables.

**Results:**

Two multisensory perception patterns were identified, representing integrated and constrained multisensory comfort. Participants belonging to the integrated pattern reported significantly higher perceived restoration than those in the constrained pattern. Positive affect was positively associated with perceived restoration, whereas negative affect showed a negative association. Among the measured environmental variables, only relative humidity exhibited a small but significant association with perceived restoration.

**Discussion:**

The findings suggest that multisensory environmental perception may exhibit recurring perception patterns rather than being fully represented by independent sensory evaluations alone. Restorative experience was associated with both multisensory perception patterns and affective responses. These findings provide empirical support for adopting a multisensory and person-centered perspective when investigating restorative environmental experiences and may inform the design of healthier and more restorative urban public spaces.

## 1 Introduction

Urbanization is reshaping the environmental conditions under which people live, work, and recover. As cities expand and densify, residents are increasingly exposed to complex environmental stimuli including fluctuating microclimates, traffic noise, artificial lighting, and visually dense built forms. These conditions impose a continuous sensory load that can accumulate into cognitive fatigue and psychological stress. At a global scale, environmental exposures in urban areas are now recognized as key determinants of public health. Large scale epidemiological evidence indicates that temperature variability and extreme heat significantly contribute to urban mortality and health risks (1, 2). At the same time, a growing body of research in environmental health demonstrates that everyday environmental experiences influence emotional regulation, stress recovery, and mental wellbeing (3, 4).

Within this context, urban green and open spaces have received increasing attention as potential restorative environments. Evidence from neuroscience shows that exposure to natural environments can reduce rumination and decrease neural activity in brain regions associated with stress (4). Population level studies further indicate that long term exposure to residential green space is associated with lower risks of psychiatric disorders (5), while longitudinal evdence suggests that individuals moving to greener urban environments experience measurable improvements in mental health (6). These findings have positioned environmental exposure as a critical component of urban health strategies and nature based solutions (3, 4, 7).

The relationship between environmental exposure and psychological recovery has been extensively examined through restoration theory. Attention Restoration Theory proposes that environments characterized by fascination, compatibility, and extent enable the recovery of depleted attentional resources (8). Stress reduction theory, in contrast, emphasizes affective responses and suggests that exposure to non threatening environments rapidly reduces physiological stress and promotes positive emotional states (9). Despite differences in emphasis, both frameworks converge on a central insight: restoration depends not only on objective environmental conditions but also on how those conditions are perceived and interpreted (10).

Recent research has extended restoration theory into contemporary urban contexts by emphasizing the role of perception in mediating environmental effects. In high density environments, environmental stress is not solely determined by physical conditions but emerges through subjective appraisal processes. Luo and Jiang (11) suggest that perceived environmental oppressiveness arises from the interaction between environmental exposure and individual evaluation, highlighting perception as a key mechanism linking environment and psychological outcomes. Understanding how individuals perceive complex urban environments is therefore essential for identifying conditions that support restoration.

However, much of the empirical literature on restorative environments has focused primarily on visual characteristics. Studies of urban parks and landscapes demonstrate that vegetation density, spatial enclosure, and landscape configuration influence perceived restorativeness (12, 13). While visual attributes are undoubtedly important, outdoor environments are inherently multisensory. Individuals simultaneously experience thermal conditions, acoustic environments, and visual landscapes when interacting with urban spaces.

Despite this, research on environmental comfort has traditionally developed along separate disciplinary trajectories. Thermal comfort studies have focused on microclimatic conditions and physiological and behavioral adaptation, while field based studies further demonstrate that outdoor comfort is shaped by contextual experience and expectation rather than by physical variables alone (14–19). Recent empirical work based on urban parks and public spaces also highlights that perceived comfort varies across users even under similar environmental conditions, reflecting the subjective nature of environmental appraisal (20).

Growing evidence suggests that such separation may overlook important interactions between sensory modalities. Experimental and field studies indicate that cross modal interactions can influence environmental appraisal and restorative perception. For example, audiovisual environmental interactions have been shown to affect perceived restoration in cold environments (21). Studies conducted in severe cold cities further demonstrate that visual, thermal, and acoustic comfort jointly shape visitors' restorative experiences (22). In addition, cross modal perception research indicates that visual environmental cues can significantly influence thermal sensation and overall environmental evaluation (23). Related studies on soundscape and environmental perception also suggest that acoustic quality interacts with visual and thermal conditions to influence overall environmental experience, highlighting the integrative nature of multisensory perception.

Nevertheless, most empirical studies continue to adopt variable centered approaches in which thermal, visual, and acoustic comfort are treated as independent predictors of restoration. Such approaches implicitly assume that environmental experience can be represented as the additive combination of separate sensory dimensions (24, 25). However, individuals exposed to similar environmental conditions may experience them in qualitatively different ways. These combinations may reflect recurring multisensory perception patterns that are not fully captured by additive models.

A configuration based perspective provides an alternative approach. Instead of focusing on the independent effects of variables, this perspective emphasizes patterns of co-occurring sensory experiences within individuals. From the perspective of restoration theory, environmental assessment emerges through the integrated interpretation of multiple environmental stimuli rather than isolated sensory responses. Individuals exposed to similar environmental conditions may therefore experience them in qualitatively different ways depending on how sensory information is jointly perceived and interpreted. To operationalize this perspective, Latent profile analysis (LPA) is employed as a person-centered statistical approach that identifies unobserved subgroups within population based on shared response patterns across multiple observed variables (26–28). Unlike conventional variable-centered methods, LPA enables multisensory environmental perception to be examined as broader multisensory perception patterns rather than independent sensory dimensions. Recent applications of person centered approaches in environmental psychology further demonstrate their potential to reveal hidden heterogeneity in environmental experience and behavioral response, supporting the use of configuration based methods in complex environmental contexts (29).

In addition to perceptual configurations, emotional responses represent a key psychological dimension that may influence restoration outcomes. Stress Reduction Theory (SRT) explicitly highlights the role of affective processes in environmental recovery (9). Emotional states such as positive affect and negative affect may shape how individuals interpret environmental conditions and, consequently, how restoration is experienced. Empirical studies in urban environments also suggest that emotional responses are closely associated with environmental perception and satisfaction, indicating that affective processes should be considered when examining restoration outcomes (20).

Seasonal context further complicates this relationship. While much of the literature focuses on extreme thermal conditions, everyday environmental experiences often occur under transitional seasonal conditions. During autumn and winter transition in subtropical urban contexts, outdoor thermal exposure may remain variable but is often less consistently dominated by sustained extreme heat or cold than during peak seasonal periods. This transitional context provides an opportunity to examine how thermal, visual, and acoustic perception jointly related to environmental assessment. Evidence from cold climate studies suggests that multisensory interactions remain important under such conditions (20, 21, 30, 31), yet empirical investigations remain limited. Rather than representing all seasonal environmental conditions, the present study focuses on a transitional climatic context in which interactions among multiple sensory dimensions may be more readily observed.

To address these gaps, the present study investigates multisensory environmental perception and psychological restoration in an urban lakeside park during the autumn and winter transitional season. A configuration based analytical approach is adopted, in which LPA is used to identify distinct multisensory comfort profiles. These profiles are subsequently used to explain differences in perceived restoration using regression models, while controlling for emotional states and measured environmental conditions.

To better examine multisensory environmental assessment under conditions where thermal stress is present but not overwhelmingly dominant, this study focuses on the autumn-winter transitional period in a subtropical urban context. Under such moderate transitional conditions, thermal discomfort is less likely to override other perceptual dimensions, thereby providing a suitable context for investigating the combined roles of thermal, visual, and acoustic perception in restorative environmental experience. Objective environmental exposure in this study was characterized using air temperature (*T*_*a*_), globe temperature (*T*_*g*_), relative humidity (*RH*) and wind speed (*v*), which together describe the microclimatic conditions under which multisensory environmental perceptions are formed. By integrating multisensory perception, affective responses, and configuration based analysis, this study contributes to a more comprehensive understanding of environmental experience in urban public spaces and provides empirical evidence to inform climate responsive and health oriented urban design.

**Figure 1:**
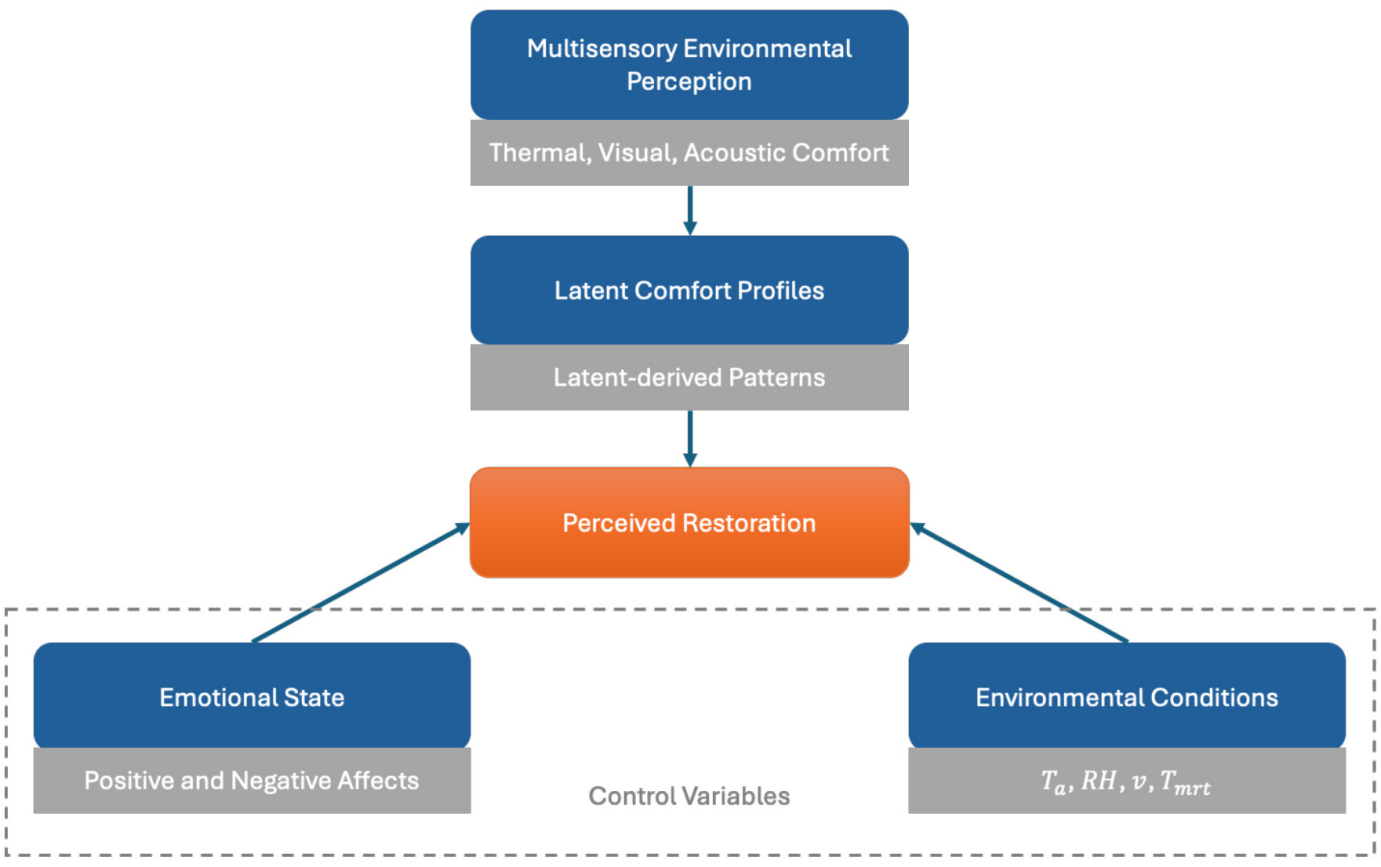
Conceputal framework of multisensory environmental perception and perceived restoration.

The conceptual framework of the study is presented in Figure 1, which conceptualizes multisensory environmental perception as latent comfort profiles and delineates their relationship with perceived restoration under the influence of emotional and environmental conditions. Based on this framework, the study proposes 3 hypothese:

1. H1: Multisensory environmental perception may exhibit recurring perceptual patterns across individuals rather than being fully explained by independent sensory evaluations alone.
2. H2: Different multisensory perception patterns may be associatated with different levels of perceived restoration.
3. H3: Affective responses may be associated with perceived restoration alongside multisensory perception patterns.

## 2 Methodology

**Figure 2:**
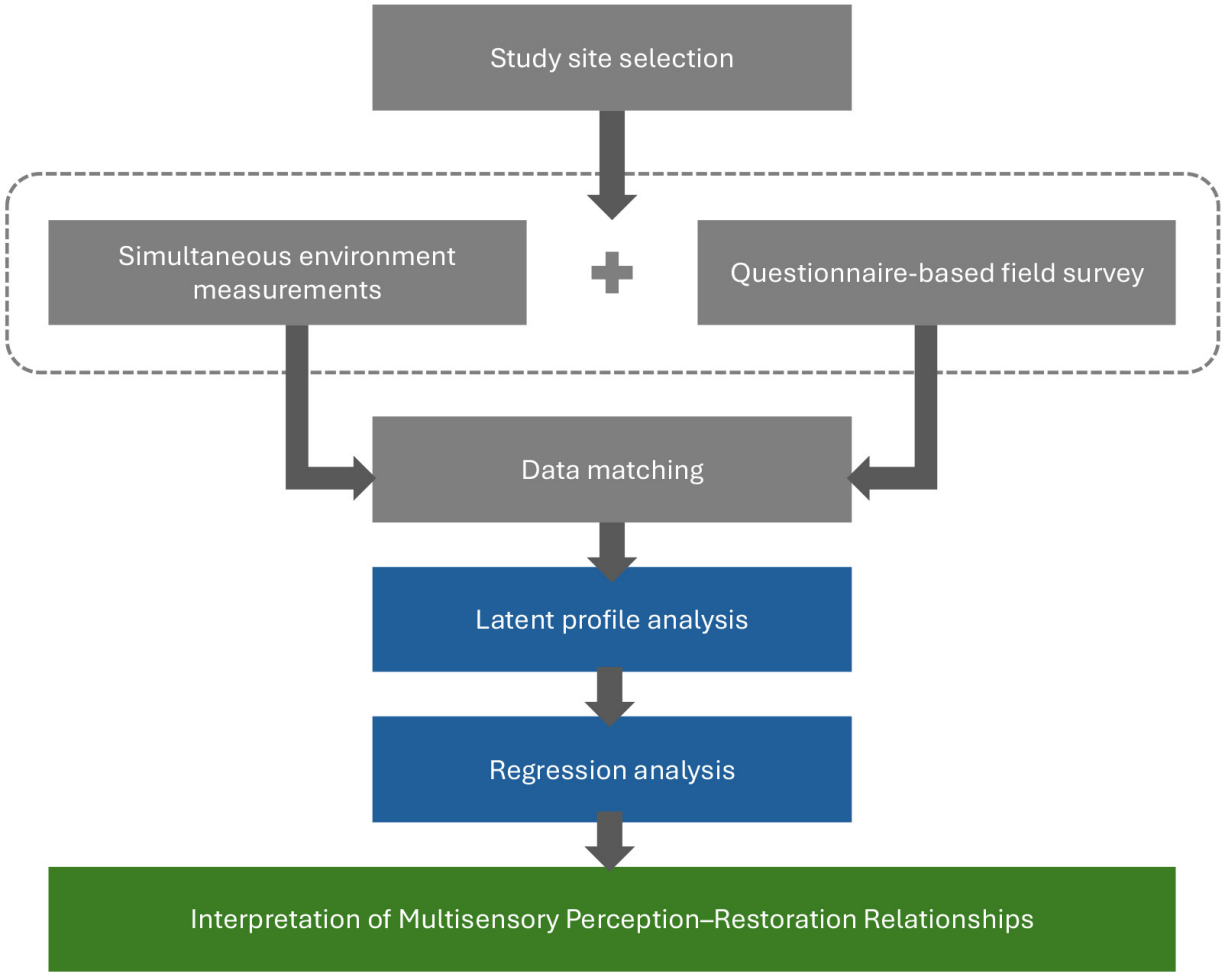
Overall study design and analytical workflow of the field survey and data analysis.

To investigate the relationship between multisensory environmental perception and perceived restoration in urban public spaces, this study combined *in situ* environmental measurements with questionnaire-based field surveys conducted in a lakeside urban park. Objective environmental conditions were recorded simultaneously with participants' subjective evaluations of thermal, visual, and acoustic comfort, affective responses, and perceived restoration. The collected data were subsequently analyzed using LPA and multiple linear regression models to examine the configuration of multisensory perception and its association with restorative experience. The overall study design and analytical workflow are illustrated in Figure 2.

### 2.1 Study site and field survey

This study was conducted in Houhu Park, an urban lakeside park located in < city>Changsha < /city>, China. The park is situated within a mixed urban environment that includes university campuses, residential neighborhoods, and clusters of knowledge based and creative workplaces such as architectural design studios, digital media companies, and game development firms. This diversity of surrounding land uses attracts a wide range of park users, including students, office workers, and local residents, making the site suitable for examining environmental experience under everyday urban conditions.

**Figure 3:**
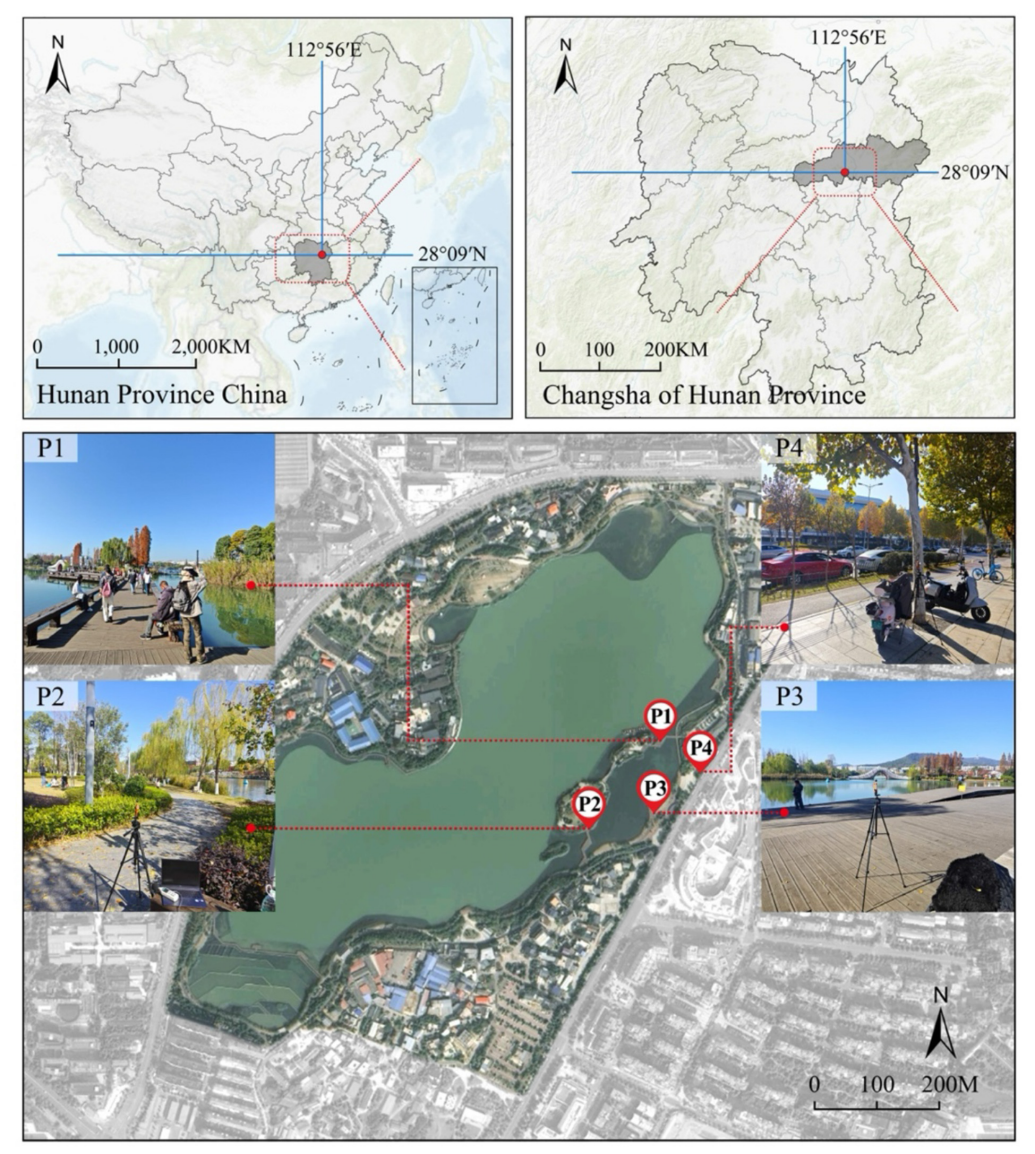
Study area and survey and field measurement locations.

The park is organized around a central water body and includes pedestrian pathways, vegetated areas, and lakeside seating spaces. Visitors moving through the park are simultaneously exposed to multiple environmental stimuli, including thermal conditions, visual landscape features, and ambient acoustic environments. The spatial context of the study area, with the locations of environmental measurement points (P1-P4) and representative site conditions, is illustrated in Figure 3. The selected points differed in vegetation density, openness, solar exposure, visual enclosure, and proximity to surrounding urban elements, thereby representing diverse environmental contexts experienced by park users. This selection strategy was intended to reflect the environmental heterogeneity of the study sites rather than focusing on a single homogeneous spatial setting.

The questionnaire was designed to capture multisensory environmental perception, perceived restoration, and emotional responses. Multisensory perception was assessed using three perceived comfort dimensions, including thermal comfort, visual comfort, and acoustic comfort, representing the primary sensory modalities through which outdoor environments are experienced (14, 30, 32). Participants evaluated each comfort dimension using a 7-point Likert scale ranging from−3 (“very uncomfortable”) to 3 (“very comfortable”). Perceived restoration was measured using items derived from PRS-11 version of the Perceived Restorativeness Scale (PRS), which operationalizes key components of restoration theory, including being away, fascination, compatibility, and extent (33–35). A composite PRS score was calculated as the mean value of all items. Emotional states were assessed using the Positive and Negative Affect Schedule (PANAS), which measures positive affect and negative affect as two independent dimensions using self-reported Likert-scale responses (36). Mean scores for both affective dimensions were computed. Demographic variables, including gender and age, were also collected and included as control variables.

Field surveys were conducted during the autumn and winter transitional season, when environmental conditions were generally moderate but variable. Participants were recruited on site after spending time within the park environment and were invited to complete a structured questionnaire immediately following their environmental experience. Before the survey, participants were asked about the duration of their stay within the park, and individuals who had only just entered the park were not approached for participation. This procedure was intended to ensure that participants had sufficient exposure to the surrounding environmental conditions before reporting their perceptual and affective responses. This *in situ* approach has been widely adopted in environmental perception and outdoor comfort research to capture real time responses (18, 37). After data screening, a total of 261 valid responses were retained for analysis.

### 2.2 Environmental measurements

**Figure 4:**
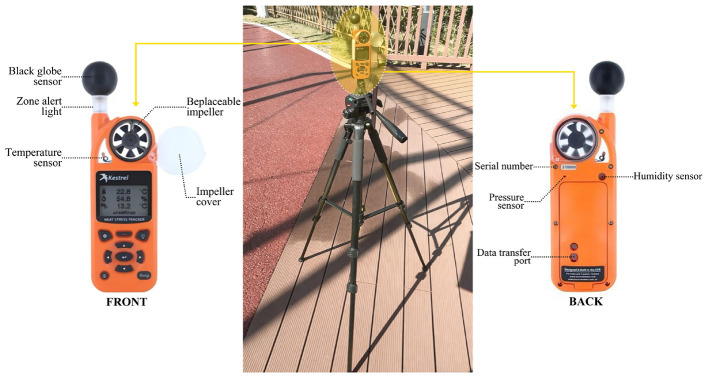
Environmental measurement instrument and field deployment.

Environmental measurements were conducted concurrently with the questionnaire survey across different period of the day to capture variations in real-world environmental exposure conditions experienced by park users. Air temperature (*T*_*a*_), globe temperature (*T*_*g*_), relative humidity (*RH*) and wind speed (*v*) at pedestrian level were measured using a portable microclimate monitoring device (Kestrel 5,400 Heat Stress Tracker). These parameters are widely used in outdoor thermal comfort research to characterize environmental exposure conditions (38). Measurements were carried out at approximately 1.1m above ground level, corresponding to the center of gravity of a standing human body, in accordance with standards for thermal environment measurements (39). Field measurements and questionnaire surveys were conducted concurrently during the daily observation period from 10:00 to 18:00, and environmental data were matched to individual questionnaire responses according to the corresponding survey time and location. The monitoring device and its field deployment are shown in Figure 4, and the specifications of sensors are summarized in Table 1.

**Table 1:** Specifications of sensors in microclimate monitoring device.

Parameter	Accuracy	Resolution	Range
*T*_*a*_	±0.5*C*	0.1*C*	−29.0 *to* 70.0*C*
*T*_*g*_	±1.4*C*	0.1*C*	−29.0 *to* 60.0*C*
*RH*	±2%	0.1%	10 − 90%
*v*	$\pm 0.1\frac{m}{s}$	$0.1\frac{m}{s}$	$0.6-14\frac{m}{s}$

The monitoring equipment is specifically designed for portable outdoor microclimate assessment and allows simultaneous measurement of key thermal environmental parameters relevant to pedestrian-level exposure. The sensor specifications summarized in Table 1 indicate that the instruments provide sufficient accuracy and temporal resolution for *in situ* environmental monitoring under dynamic outdoor conditions. To provide a physiologically informed characterization of outdoor thermal stress, the Universal Thermal Climate Index (UTCI) was additionally calculated based on the measured air temperature, relative humidity, wind speed, and mean radiant temperature. UTCI was included as a contextual descriptor of the thermal exposure conditions observed during the field survey.

Radiative exchange was not directly measured. Instead, globe temperature (*T*_*g*_) was used as an integrated indicator reflecting the combined effects of shortwave and longwave radiation. Based on this, mean radiant temperature (*T*_*mrt*_) was calculated using *T*_*a*_, *T*_*g*_, and *v* following established formulations in outdoor thermal comfort research (40):


$$\begin{array}{l} T_{mrt}={\left[{\left(T_{g}+273.15\right)}^{4}+\frac{1.10\times 10^{8}v_{a}^{0.6}}{\zeta D^{0.4}}\left(T_{g}-T_{a}\right)\right]}^{\frac{1}{4}}-273.15 \end{array}$$ (1)

where *T*_*g*_ is globe temperature (*C*), *T*_*a*_ is air temperature (*C*), and *v*_*a*_ is wind speed ($\frac{m}{s}$). ζ represents the emissivity of the globe, and *D* denotes the globe diameter (*m*). In this study, a standard black globe with an emissivity of 0.95 and a diameter of 0.15 m was used (40).

### 2.3 Data analysis

The analytical framework of this study aimed to identify patterns of multisensory environmental perception and examine their relationship with perceived restoration while accounting for emontional and environmental conditions. Prior to statistical analysis, incomplete questionnaires and responses with missing information on key study variables were excluded to ensure data quality and consistency in the LPA and regression procedures.

First, descriptive statistical analyses were conducted to summarize the distributions of multisensory comfort variables, perceived restoration scores, and emotional responses. These analyses provide an overview of participants' environmental perceptions and psychological experiences during the field survey.

To identify patterns of multisensory environmental perception, LPA was applied using participants' reported levels of thermal comfort, visual comfort, and acoustic comfort. LPA is a person-centered statistical approach used to identify unobserved subgroups within a population based on responses across multiple continuous variables (41). The method belongs to the broader class of finite mixture models, which assume that the observed data are generated from a mixture of latent subpopulations (42). Formally, the probability distribution of the observed data can be expressed as:


$$\begin{array}{l} f\left(y_{i}\right)=\overset{K}{\underset{k=1}{\sum }}\pi_{k}f_{k}\left(y_{i}|\theta_{k}\right) \end{array}$$ (2)

where *K* represents the number of latent profiles, π_*k*_ denotes the probability of belonging to profile *k*, and *f*_*k*_(*y*_*i*_|θ_*k*_) represents the profile-specific distribution of the observed response vector *y*_*i*_ given parameter set θ_*k*_.

The posterior probability that individual *i* belongs to latent profile *k* can be estimated using Bayes' theorem:


$$\begin{array}{l} P\left(z_{i}=k|y_{i}\right)=\frac{\pi_{k}f_{k}\left(y_{i}|\theta_{k}\right)}{\overset{K}{\underset{j=1}{\sum }}\pi_{j}f_{j}\left(y_{i}|\theta_{j}\right)} \end{array}$$ (3)

where *P*(*z*_*i*_ = *k*|*y*_*i*_) represents the posterior probability of assigning individual *i* to profile *k*, and the denominator represents the total probability across all profiles. Each participant was assigned to the profile with the highest posterior probability following a maximum-probability classification rule. The mean posterior probability was used to assess classification quality, with higher values indicating clearer separation between profiles.

Models with increasing numbers of profiles were estimated and compared using Akaike Information Criterion (AIC), Bayesian Information Criterion (BIC), and classification performance indicators. The optimal number of profiles was determined based on model fit, parsimony, and interpretability, following established recommendations (43).

To examine the association between multisensory comfort profiles and perceived restoration, an ordinary least squares (OLS) regression model was estimated. Profile membership was included as a dummy variable, with the constrained profile serving as the reference group. The model can be expressed as:


$$\begin{array}{lll} PRS_{i} & = & \beta_{0}+\beta_{1}Profile_{i}+\beta_{2}PA_{i}+\beta_{3}NA_{i}+\beta_{4}Gender_{i} \end{array}$$

$$\begin{array}{l} +\;\beta_{5}Age_{i}+\beta_{6}{T_{a}}_{i}+\beta_{7}RH_{i}+\beta_{8}v_{i}+\beta_{2}{T_{mrt}}_{i}+\varepsilon_{i} \end{array}$$ (4)

where *PRS*_*i*_ represents perceived restoration, *Profile*_*i*_ indicates latent profile membership, and *PA*_*i*_ and *NA*_*i*_ denote positive affect and negative affect, respectively. β_0_ is the intercept, and β_1_ to β_9_ represent the regression coefficients of the corresponding predictors. Demographic variables, including gender and age, and environmental variables, including *T*_*a*_, RH, v, and *T*_*mrt*_, were included as control variables. ε_*i*_ denotes the random error term.

## 3 Results

The sample consisted of 61.7% female and 38.3% male respondents, with a mean age of 23.6 years (SD = 5.3), reflecting that the study primarily captured responses from a young adult population.

The environmental conditions recorded during the field survey are summarized in Table 2. Air temperature ranged from 11.9 °C to 33.3 °C, with a mean of 20.0 °C (SD=4.4 °C), covering a relative broad thermal range. Globe temperature varied between 11.0 °C and 43.9 °C, showing a wider spread than air temperature and indicating variations in radiative exposure. Relative humidity ranged from 26.4% to 84.6%, demonstrating substantial fluctuation over the observation period. Wind speed remained generally low, ranging from 0.0 to 4.5m/s. The UTCI ranged from 3.0 °C to 37.3 °C, with a mean of 22.6 °C (SD = 6.0 °C), further indicating that participants were exposed to a relatively broad range of outdoor thermal conditions during the survey period.

**Table 2:** Descriptive statistics of microclimatic parameters during the study period.

Parameter	Min	Max	Mean	SD
*T*_*a*_	11.9*C*	33.3*C*	20.0*C*	3.8*C*
*T*_*g*_	11.0*C*	43.9*C*	24.5*C*	6.5*C*
*RH*	26.4%	84.6%	47.8%	12.8%
*v*	$0.0\frac{m}{s}$	$4.5\frac{m}{s}$	$0.7\frac{m}{s}$	$0.7\frac{m}{s}$
*T*_*mrt*_	7.8*C*	83.7*C*	32.3*C*	14.4*C*
UTCI	3.0*C*	37.3*C*	22.6*C*	6.0*C*

Across the monitored parameters, variability was observed throughout the survey period rather than stable conditions. This variability indicates that participants experienced different environmental conditions across the survey period, which may have contributed to differences in multisensory environmental perception. Differences in dispersion were also apparent across variables, with globe temperature and mean radiant temperature showing greater variability compared with air temperature and wind velocity. These measurements together describe a dynamic but non-uniform environmental context during the field survey.

Reliability analysis was further conducted for the principal psychological measures used in the study, and the results are summarized in Table 3.

**Table 3:** Reliability statistics of psychological scales.

Scale	Number of items	Cronbach's α
PRS	11	0.840
PA	10	0.746
NA	10	0.901

LPA was conducted to identify patterns of multisensory environmental perception, and the model fit indices are presented in Table 4. The one-profile solution yielded an AIC of 2,352.679 and a BIC of 2,384.759. Introducing a second profile resulted in a marked reduction in both AIC and BIC, representing a clear improvement in model fit. With additional profiles, AIC continued to decrease and reached 2,261.173 in the five-profile solution. In contrast, BIC increased beyond the two-profile model, reaching 2,393.669 for three profiles, 2,420.544 for four profiles, and 2,435.834 for five profiles. The mean posterior probability for the two-profile solution was 0.891, reflecting a high level of classification precision. Models with more profiles produced comparable posterior probabilities but did not show clear gains in classification performance. In addition, the additional profile solutions resulted in less interpretable profile structures and smaller incremental improvements in model fit. Considering the combined evidence from model fit indices and classification accuracy, the two-profile solution was retained for subsequent analysis.

**Table 4:** Model fit statistics for latent profile models of multisensory comfort.

Profiles	AIC	BIC	Mean max posterior probability
1	2,352.679	2,384.759	1.000
2	2,289.350	2,357.076	0.891
3	2,290.298	2,393.669	0.836
4	2,281.528	2,420.544	0.850
5	2,261.173	2,435.834	0.871
6	2,280.551	2,490.858	0.872

The characteristics of the identified profiles are summarized in Table 5. The integrated multisensory comfort profile comprised 157 participants (60.2% of the sample) and was associated with relatively high comfort scores across all sensory dimensions, with mean values of 5.726 for thermal comfort, 5.841 for visual comfort, and 4.662 for acoustic comfort. The constrained multisensory comfort profile included 104 participants (39.8%) and showed lower comfort levels overall, with mean values of 4.298 for thermal comfort, 3.856 for visual comfort, and 4.106 for acoustic comfort.

**Table 5:** Characteristics of multisensory environmental perception profiles.

Profile	Thermal comfort	Visual comfort	Acoustic comfort
Constrained	4.298	3.856	4.106
Integrated	5.726	5.841	4.662

**Figure 5:**
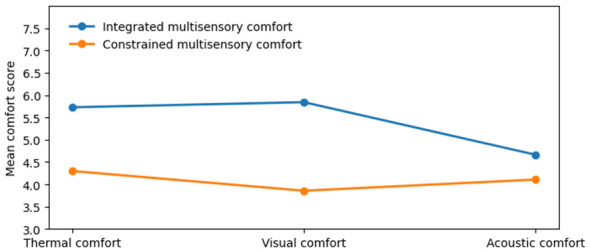
Multisensory environmental comfort profiles identified by LPA.

The magnitude of difference between the two profiles varies across sensory dimensions. The largest difference is observed in the visual comfort, followed by thermal comfort, while acoustic comfort shows comparatively smaller variation. As shown in Figure 5, the integrated profile reflects a relatively balanced and elevated multisensory experience, whereas the constrained profile is characterized by reduced visual comfort and generally lower overall satisfaction. This pattern indicates that visual and thermal perceptions play a dominant role in structuring multisensory experience, whereas acoustic perception contributes less to distinguishing perceptual configurations in this context. These findings suggest that multisensory environmental perception exhibits recurring multisensory perception patterns rather than independent sensory responses alone, with visual and thermal dimensions playing a more prominent role in differentiating perceptual profiles.

Correlation analysis indicated that positive affect and negative affect were only weakly correlated (*r* = 0.168). Potential multicollinearity among regression predictors was further assessed using variance inflation factors (VIFs), and all predictor VIF values were below 2, indicating no evidence of problematic multicollinearity.

Regression results are presented in Tables 6, 7. The model explained 36.3% of the variance in perceived restoration. Profile membership shows a statistically significant positive association with perceived restoration, with higher restoration scores observed among participants in integrated multisensory comfort profile.

**Table 6:** Model fit statistics.

R^2^	Adjusted R^2^
0.363	0.340

**Table 7:** Regression results.

Variable	β	S.E.	*p*-value
*Intercept*	4.557	0.552	0.000
*Profile* (Constrained = 0)	0.520	0.099	0.000
*PA*	0.560	0.077	0.000
*NA*	−0.184	0.072	0.011
*Gender* (male=0)	0.016	0.097	0.873
*Age*	−0.011	0.009	0.243
*T*_*a*_	−0.012	0.017	0.490
*RH*	−0.009	0.004	0.026
*v*	−0.097	0.117	0.407
*T*_*mrt*_	0.004	0.004	0.329

Positive affect (PA) is positively associated with perceived restoration, while negative affects (NA) show a significant negative relationship. Among the environmental variables, only relative humidity is statistically significant, with a small negative effect on perceived restoration. Other environmental variables, including air temperature, wind speed, and mean radiant temperature, do not show significant associations. Demographic variables, including gender and age, are not significant predictors.

## 4 Discussion

A central observation is that participants were grouped into distinct multisensory perception profiles rather than distributed along a single continuum of comfort. This suggests that environmental experience may exhibit recurring multisensory perception patterns that cannot be fully explained by independent sensory dimensions alone. Such findings suggest that additive models alone may not fully capture the complexity of multisensory environment perception, where thermal, visual, and acoustic dimensions are assumed to operate independently. Instead, the findings indicate that multiple sensory dimensions may be jointly reflected in broader perceptual evaluations of environmental experience. This interpretation is consistent with recent advances in multisensory environmental research, which emphasize that perception emerges from the interaction of sensory modalities rather than from isolated sensory channels (21, 44). It also aligns with broader perspectives in environmental psychology that highlight the role of subjective appraisal in shaping environmental experience (11).

The relative importance of different sensory dimensions also provides insight into how perceptual configurations are formed. Visual comfort contributed most strongly to profile differentiation, followed by thermal comfort, while acoustic differences were comparatively smaller. Previous studies have shown that visual environmental characteristics, such as landscape structure and openness, play a dominant role in shaping environmental preference and perceived restorativeness (7, 45, 46). At the same time, thermal perception has been shown to vary with both environmental conditions and individual adaptation (14). Acoustic perception, in contrast, may be more sensitive to contextual variability in sound sources and less influential when environmental noise levels remain relatively stable (47). The differences suggest that the salience of sensory modalities in shaping environmental perception is context-dependent and may vary across environmental settings.

Another key finding concerns the strong association between affective responses and perceived restoration. Positive affect showed a substantial relationship with restoration, while negative affect was negatively associated. This pattern supports theoretical frameworks that emphasize the role of affect in restorative processes. SRT proposes that exposure to non-threatening environments leads to rapid affective responses, which in turn facilitate recovery from stress (9). Empirical studies have similarly demonstrated that exposure to natural environments is associated with reduced negative affect and enhanced positive emotional states (4, 6, 48). The present findings are consistent with this body of work, suggesting that affective responses are not merely by-products of environmental exposure but are central to how restorative outcomes are experienced. At the same time, the independent contribution of multisensory perception indicates that perceptual appraisal and affective response represent related but distinct processes.

In contrast, objective environmental variables showed limited direct effects. With the exception of relative humidity, most physical parameters were not significantly associated with perceived restoration. Similar findings have been reported in studies where subjective perception was found to be more strongly related to environmental evaluation than objective measurements (49). One possible explanation is that, across much of the observed environmental range during the winter-autumn transitional period, physical stressors may not have consistently reached thresholds sufficient to influence restorative experience directly. Instead, their influence may operate indirectly through perceptual and affective pathways. This interpretation is consistent with research suggesting that environmental effects on wellbeing are mediated by subjective appraisal processes (11).

Several contextual factors should be considered when interpreting the present findings. Because the field survey was conducted during the autumn–winter transitional period under relatively moderate outdoor thermal conditions, the identified multisensory perception profiles may reflect perceptual structures specific to this climatic context. Under more thermally extreme summer or winter conditions, where thermal stress exerts a stronger influence on environmental assessment, different multisensory configurations may emerge. Because data were collected under variable environmental conditions across the survey period, some differences in latent profile classification may partially reflect temporal and environmental variability rather than purely stable perceptual tendencies. In addition, the sample was predominantly composed of young adult park users, which may constrain the generalizability of the findings to broader demographic groups and socio-cultural populations. Individuals who choose to visit urban parks may already possess relatively positive environmental preferences or restorative expectations. Future research should therefore examine whether similar perceptual patterns can be observed across different age groups, socio-cultural contexts, climatic conditions, and urban environmental settings. In addition, because the study adopted a cross-sectional field design, the observed relationships should be interpreted as associative rather than causal.

These findings suggest that restorative experience is associated with the interplay between environmental exposure, multisensory perception patterns, and affective response. This supports an integrative perspective in which environmental conditions were associated with perception, perception interacts with affect, and these processes jointly shape restorative outcomes. Such a framework contributes to existing restoration research by incorporating multisensory perception and person-centered analysis into the understanding of environmental experience.

## 5 Conclusion

This study examined how multisensory environmental perception relates to perceived restoration in outdoor urban settings. The findings show that individuals may not evaluate environmental conditions solely as isolated sensory dimensions but instead exhibit multisensory perception patterns. These configurations are associated with differences in perceived restoration.

The results further demonstrate that both multisensory perception and affective responses contribute to restorative experience, while objective environmental variables showed limited direct associations within the observed range of transitional outdoor conditions. This highlights the importance of considering both perceptual and emotional processes in understanding environmental effects on wellbeing.

This study makes three main contributions. First, it introduces a configuration-based perspective on multisensory environmental perception, moving beyond traditional additive approaches. Second, it clarifies the complementary roles of perception and affect in shaping restoration. Third, it provides field-based evidence under transitional seasonal conditions, expanding current research beyond extreme environments.

These findings suggest that designing restorative urban environments requires attention to the overall coherence of multisensory experience rather than focusing on single environmental factors. Future research should further explore how perceptual configurations are formed across different populations and environmental contexts, and how they can be incorporated into predictive models of environmental experience.

## Data Availability

The raw data supporting the conclusions of this article will be made available by the authors, without undue reservation.
